# Supplementary material for: Factors Associated With Mental Health Outcomes Among Health Care Workers Exposed to Coronavirus Disease 2019
Source: JAMA Netw Open. 2020 Mar 23;3(3):e203976. doi: 10.1001/jamanetworkopen.2020.3976 (PMC7090843; doi:10.1001/jamanetworkopen.2020.3976)
Supplement: Supplement. — eFigure. Milestone Events During the Outbreak and Epidemic of COVID-19 eTable 1. Occupation and Geographic Data of Nonrespondents eTable 2. Intergroup Comparisons of Regional Differences on Scores of Depression, Anxiety, Insomnia, and Distress eTable 3. Factor Scores of IES-R in Total Participants and Subgroups eTable 4. Hospital Type and Factor Scores of IES-R in Front-line Workers eTable 5. Intergroup Comparisons of Regional Differences on Factor Scores of IES-R [file jamanetwopen-3-e203976-s001.pdf]

Supplementary Online Content

Lai J, Ma S, Wang Y, et al. Factors associated with mental health outcomes among health care workers exposed to coronavirus disease 2019. *JAMA Netw Open*. 2020;3(3):e203976. doi:10.1001/jamanetworkopen.2020.3976

eFigure. Milestone Events During the Outbreak and Epidemic of COVID-19
eTable 1. Occupation and Geographic Data of Nonrespondents
eTable 2. Intergroup Comparisons of Regional Differences on Scores of Depression, Anxiety, Insomnia, and Distress
eTable 3. Factor Scores of IES-R in Total Participants and Subgroups
eTable 4. Hospital Type and Factor Scores of IES-R in Front-line Workers
eTable 5. Intergroup Comparisons of Regional Differences on Factor Scores of IES-R

This supplementary material has been provided by the authors to give readers additional information about their work.

© 2020 Lai J et al. *JAMA Network Open.*

eFigure. Milestone Events during the Outbreak and Epidemic of COVID-19[a]

[a] From December 31, 2019 to January 31, 2020, the confirmed case of COVID-19 increased dramatically from 27 to over 10,000. This study was conducted from January 29 to February 3, 2020, as indicated in the figure.

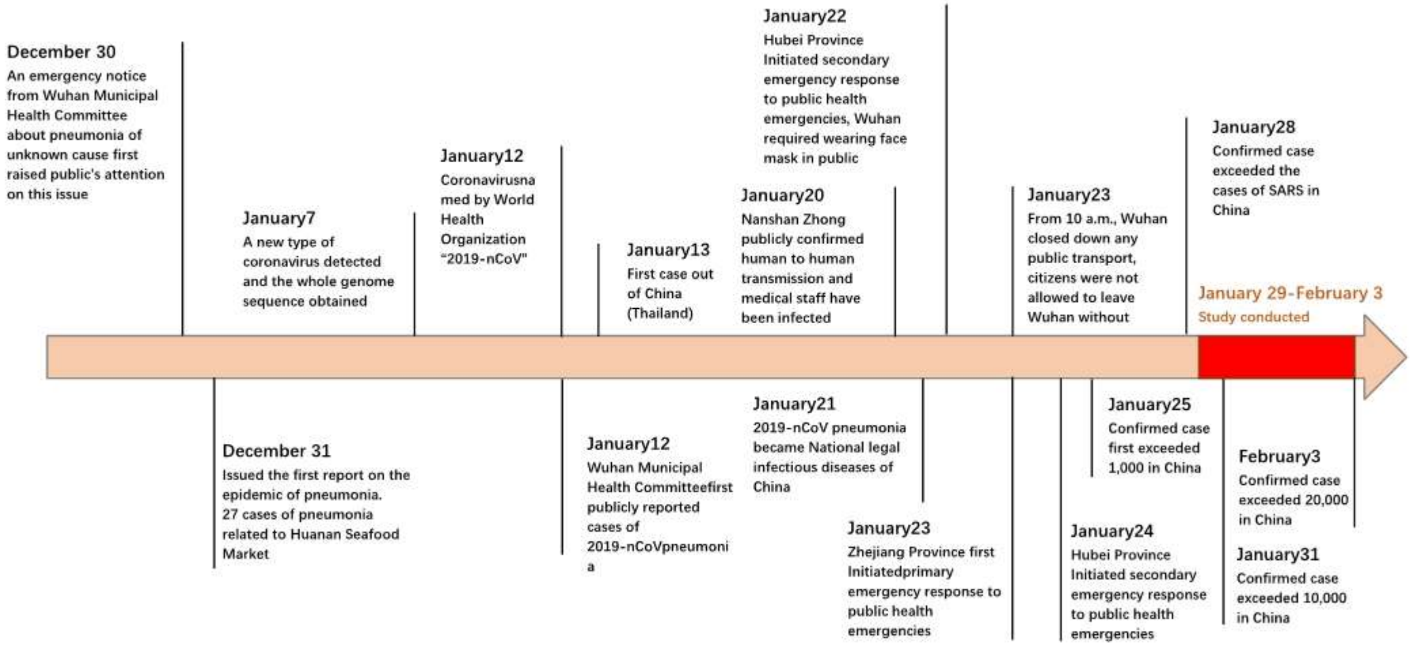

© 2020 Lai J et al. *JAMA Network Open.*

eTable 1. Occupation and Geographic Data of Nonrespondents

	Total[a]	Occupation		Geographic location		
		Physician	Nurse	Wuhan	Hubei Province outside of Wuhan)	Outside Hubei Province
No, (%)	573	209(36.5)	364(63.5)	349(60.9)	111(19.4)	113(19.7)

[a] Among the 1830 individuals asked to participate, 573 (31.3%) did not respond to the survey.

© 2020 Lai J et al. *JAMA Network Open.*

eTable 2. Intergroup Comparisons of Regional Differences on Scores of Symptoms of Depression, Anxiety, Insomnia, and Distress

Scale	Geographic location			*P1*[a]	*P2*[b]	*P3*[c]
	Wuhan	Hubei Province outside ofWuhan	Outside Hubei Province			
PHQ-9, median (IQR)	5.0(2.0, 8.0)	4.0(1.0, 7.0)	3.0(0.0, 7.0)	.42	.02	< .001
GAD-7, median (IQR)	4.0(1.0, 7.0)	3.0(0.0, 6.0)	2.0(0.0, 6.0)	.40	.01	< .001
ISI, median (IQR)	5.0(2.0, 10.0)	4.0(1.0, 8.0)	3.0(1.0, 8.0)	.65	.02	< .001
IES-R, median (IQR)	21.0(8.5, 34.5)	18.0(6.0, 28.0)	15.0(4.0, 26.0)	.34	.007	< .001

Abbreviation: PHQ-9, 9-item Patient Health Questionnaire; GAD-7, 7-item Generalized Anxiety Disorder; ISI, 7-item Insomnia Severity Index; IES-R, 22-item Impact of Event Scale-Revised; IQR, interquartile range.

[a] Outside Hubei Province vs Hubei Province outside of Wuhan.

[b] Wuhan vs Hubei Province outside of Wuhan.

[c] Wuhan v. Outside Hubei Province.

© 2020 Lai J et al. *JAMA Network Open.*

eTable 3. Factor Scores of IES-R in Total Participants and Subgroups

Factor	Total score	Occupation			Sex			Working position			Type of hospital		
		Physicina	Nurse	*P* value	Male	Female	*P* value	Front-line	Second-line	*P* value	Tertiary	Secondary	*P* value
Avoidance, median (IQR)	6.0(2.0, 11.0)	6.0(1.0, 10.0)	6.0(2.0, 12.0)	.02	5.0(0.0, 10.0)	7.0(2.0, 11.0)	<.001	7.0(2.0, 12.0)	5.0(1.0, 10.0)	<.001	6.0(2.0, 11.0)	6.0(1.0, 11.0)	.35
Intrusion, median (IQR)	8.0(3.0, 13.0)	7.0(2.0, 13.0)	8.0(4.0, 13.0)	.006	6.0(2.0, 11.0)	8.0(4.0, 13.0)	<.001	9.0(4.0, 14.0)	7.0(3.0, 11.0)	<.001	8.0(4.0, 13.0)	8.0(3.0, 13.0)	.47
Hyperarousal, median (IQR)	5.0(1.0, 8.0)	4.0(1.0, 8.0)	5.0(1.5, 9.0)	.02	3.0(0.0, 8.0)	5.0(2.0, 9.0)	<.001	6.0(2.0, 9.0)	4.0(1.0, 8.0)	<.001	5.0(1.0, 8.0)	5.0(1.0, 9.0)	.19

Abbreviation: IES-R, 22-item Impact of Event Scale-Revised; IQR, interquartile range.

© 2020 Lai J et al. *JAMA Network Open.*

eTable 4. Hospital Type and Factor Scores of IES-R in Front-line Workers

Factor	First-line workers		
	Tertiary hospital	Secondary hospital	*P* value
Avoidance, median (IQR)	8.0(2.0, 13.0)	6.0(2.0, 11.0)	.06
Intrusion, median (IQR)	9.0(4.0, 15.0)	8.0(4.0, 13.0)	.15
Hyperarousal, median (IQR)	6.0(2.0, 10.0)	6.0(2.0, 9.0)	.29

Abbreviation: IES-R, 22-item Impact of Event Scale-Revised; IQR, interquartile range.

© 2020 Lai J et al. *JAMA Network Open.*

eTable 5. Intergroup Comparisons of Regional Differences on Factor Scores of IES-R

Scale	Geographic location			$P1^a$	$P2^b$	$P3^c$
	Wuhan	Hubei Province outside of Wuhan)	Outside Hubei Province			
Avoidance, median (IQR)	7.0(2.0, 12.0)	5.0(2.0, 10.0)	4.0(1.0, 9.0)	.47	.01	<.001
Intrusion, median (IQR)	8.0(4.0, 14.0)	7.0(3.0, 11.0)	6.0(2.0, 11.0)	.93	.001	<.001
Hyperarousal, median (IQR)	5.0(2.0, 9.0)	5.0(1.0, 8.0)	3.0(0.0, 7.0)	.06	.19	<.001

Abbreviation: IES-R, 22-item Impact of Event Scale-Revised; IQR, interquartile range.

[a] Outside Hubei Province vs Hubei Province outide Wuhan.

[b] Wuhan vs Hubei Province outside ofWuhan.

[c] Wuhan vs Outside Hubei Province.

© 2020 Lai J et al. *JAMA Network Open.*
